# Cytogenetic Biomonitoring on Oral Mucosa Cells of Hookah Users: Is It Possible? 

**DOI:** 10.31557/APJCP.2020.21.7.1849

**Published:** 2020-07

**Authors:** Ana Carolina Flygare Souza, Marina Gomes Galvani, Daniel Vitor de Souza, Daniel Araki Ribeiro

**Affiliations:** *Department of Biosciences, Federal University of Sao Paulo, UNIFESP, SP, Brazil. *

**Dear Editor**

Genotoxicity and cytotoxicity are defined as the ability of an agent to cause injury on genetic material and to induce cellular death, respectively. These occurrences have clinical implications because DNA damage and cell death are closely involved to several diseases including cancer. Herein, this is important to understand what chemical agents induce harmful effects on oral cells under different contexts and paradigms. This information will contribute to a better understanding the pathways closely involved to genotoxicity/cytotoxicity in order to prevent diseases in the oral cavity. The manuscript recently published by Taghibakhsh et al., (2019) have assumed that Hookah use is able to induce chromosome breakage and cellular death in oral mucosa cells by means of micronucleus test. However, this study has some issues that motivates us to establish a discussion about the matter.

In Material and methods, it was not mentioned what staining process was used in the study. We supposed that Papanicolaou was made since it was described in the Abstract. However, the technique is not suitable for this purpose since it is not specific for nucleic acids. This inevitably leads to false positive results due to the identification of cell structures that resembling micronucleus, such as keratohyalin granules or bacteria (Bonassi et al., 2011). This may explain the high micronucleus frequencies found by the authors. Another question refers to the number of cells evaluated. The authors analyzed a total of 500 cells only. According to the Micronucleus Assay Expert Group, it is mandatory to evaluate a minimum of 2,000 oral cells per volunteer (Torres-Bugarin et al., 2014). To increase the total number of cells would significantly improve the power analysis of the assay.

In the manuscript, the figures illustrate some metanuclear changes, such as micronucleus, broken egg, and karyorrhexis. Nevertheless, these ones are not properly defined. For example, the micronucleus highlighted in Figure 1 does not have the same colour and texture as well as it is not the same plane of focus when compared to main nucleus. These requisites are very important for identifying the micronucleus in any eukaryotic cell. Furthermore, broken egg is not inside the cell and, finally; it was not possible to identify karyorrhexis with accuracy in Figure 2.

In the Results, the authors revealed that “As shown in Table 1, the mean of MN percentage was 1.8 folds higher in the hookah group (10.7) compared to the control group (5.8) (P<0.001), The mean percentages of KR in the control and case groups were 0.04 and 0.1, respectively, which was 2.5 folds higher in the case group compared to the control group (P<0.001). In addition, the mean percentages of KL in the control and case groups. were 0.08 and 0.16, respectively, which was 2 folds higher in the control group compared to the case group (P<0.026). The repair index in the control and test groups were 0.05 and 0.03, respectively (Table 2), which was 40% higher in the control group compared to the case group (P<0.026)”. Unfortunately, the results presented in Tables 1 and 2 are inverted as those described by the authors. This requires further clarification. 

We believe that these comments be useful for better understanding the important article investigating cytogenetic damage on oral mucosa cells of Hookah users. 

## References

[B1] Bonassi S, Coskun E, Ceppi M (2011). The HUman MicroNucleus project on eXfoLiated buccal cells (HUMN(XL)): the role of life-style, host factors, occupational exposures, health status, and assay protocol. Mutat Res.

[B2] Taghibakhsh M, Farhadi S, Babaee A, Sheikhi M (2019). The effect of hookah use on buccal mucosa: Evaluation of repair index. Asian Pac J Cancer Prev.

[B3] Torres-Bugarín O, Zavala-Cerna MG, Nava A, Flores-García A, Ramos-Ibarra ML (2014). Potential uses, limitations, and basic procedures of micronuclei and nuclear abnormalities in buccal cells. Dis Markers.

